# Cellular Signal Detection by Hydrogenated Amorphous Silicon Photosensitive Chip with Electroexcitation

**DOI:** 10.3390/s25175255

**Published:** 2025-08-23

**Authors:** Fengyan Hou, Jianjun Dong, Xia Wang, Qiuyang Deng, M. James C. Crabbe, Zuobin Wang

**Affiliations:** 1 International Research Centre for Nano Handling and Manufacturing of China, Changchun University of Science and Technology, Changchun 130022, China; 2021200036@mails.cust.edu.cn (F.H.); 2020200030@mails.cust.edu.cn (J.D.); 2021200032@mails.cust.edu.cn (X.W.); 2023200027@mails.cust.edu.cn (Q.D.); 2 Centre for Opto/Bio-Nano Measurement and Manufacturing, Zhongshan Institute of Changchun University of Science and Technology, Zhongshan 528437, China; 3 Ministry of Education Key Laboratory for Cross-Scale Micro and Nano Manufacturing, Changchun University of Science and Technology, Changchun 130022, China; 4 Wolfson College, University of Oxford, Oxford OX2 6UD, UK; james.crabbe@wolfson.ox.ac.uk; 5 IBEST & IRAC, University of Bedfordshire, Luton LU1 3JU, UK

**Keywords:** a-Si:H photosensitive chip, light-induced electrode, cellular electrical detection, cell–material interface

## Abstract

Based on the photoconductive effect of photosensitive films, a designed light pattern was projected onto a hydrogenated amorphous silicon (a-Si:H) photosensitive chip to generate virtual light-induced electrodes for cellular electrical detection. To obtain high-quality cellular signals, this study aims to explore the effect of electrical excitation on a-Si:H photosensitive chip. Firstly, the electrochemical impedance spectroscopy (EIS) and volt-ampere characteristics of the a-Si:H photosensitive chip were characterized. EIS data were fitted to extract equivalent circuit models (ECMs) for both the chip and system. Then analog experiments were performed to verify the ECMs, and the results were consistent with the circuit simulation. Finally, applied alternating current (AC) or direct current (DC) signals to the chip and recorded the electrical signals of the cultured cardiomyocytes on the a-Si:H photosensitive chip. The results demonstrated that applying a high-frequency small AC signal to the chip reduced the background noise of the system by approximately 85.1%, and applying a DC bias increased the amplitude of the detection signal by approximately 142.7%. Consequently, the detection performance of the a-Si:H photosensitive chip for weak bioelectrical signals was significantly enhanced, advancing its applicability in cellular electrophysiological studies.

## 1. Introduction

Patch clamps [1,2] and microelectrode arrays (MEAs) [3,4] are important technologies in electrophysiological research. The emergence of automatic patch clamp [5] has greatly improved the efficiency of high-throughput and standardized experiments. However, the automation of patch clamp only optimizes the stability and efficiency of operation, and still has invasiveness, which is unsuitable for long-term recordings [6]. MEAs have been widely concerned since their emergence. According to the spatial distribution of microelectrodes on the substrate, electrodes can be two-dimensional [7] or three-dimensional [8]. Depending on the properties of the substrate material, they can be divided into rigid electrodes or flexible electrodes [9]. However, MEA needs the design and preparation of complex physical electrodes [10] and is limited to recording at fixed locations. To overcome these limitations, our group recently proposed a novel cellular electrical signal detection technology, light-induced electrode scanning microscopy [11]. It is based on the photoconductive effect of photosensitive films [12], which creates localized conductive channels in the illuminated area, namely virtual light-induced electrodes. The light-induced electrodes enable continuous (no measurement blind zone), low-cost, flexible reconfiguration, and non-destructive measurements, which provide a new tool for the detection of the electrical properties of cells. The details are described in the supplementary information (References [13,14] are cited in the supplementary information). As a label-free cellular electrophysiology detection, this technology differs from biosensors [15], requiring biological recognition elements. Its innovation lies in employing programmable virtual light-induced electrodes for detection, thereby extending the capabilities of electrophysiological detection tools.

Photoelectronic devices have demonstrated distinct advantages in cell biology research through efficient conversion and precise regulation of optoelectronic signals. As a semiconductor material, a-Si:H has excellent photosensitizing properties [16] and good biocompatibility. In addition, plasma-enhanced chemical vapor deposition (PECVD) [17] can deposit a-Si:H films uniformly over a large area, having good compatibility with indium tin oxide (ITO) glass substrates. a-Si:H has been widely used in solar cells [18], photodetectors [19], optically induced dielectrophoresis [20], and biomolecular detection [21]. However, the performance of a-Si:H is limited by the intrinsic properties of the material. Due to the relatively high density of defect states in amorphous silicon [22], it is affected by a large amount of dark current. Therefore, as a cell-interface detection interface, its detection signal-to-noise ratio needs to be improved.

The extracellular electrical signals of biological cells are weak and of low frequency. The significant feature of the electrical signals is that the amplitude is only millivolts or microvolts, and the noise is high [23]. Accurately detecting and extracting cellular electrical signals is the basis for the diagnosis and prevention of various physiological diseases. It provides an important support for medical research and clinical applications. Therefore, reducing noise or amplifying signals to improve the signal-to-noise ratio is particularly important for the study of weak bioelectric signals.

Traditional approaches to improving a-Si:H performance focus on passive optimization via material modification. For instance, some researchers have attempted to solve the problem of low conductivity in a-Si:H films in terms of preparation process and structure [19,20]. In addition, by changing process parameters such as applied electricity, pressure, gas, and electrolyte concentration, or internal components such as materials, catalyst and electrolyte types, and observing impedance trends, the effects of these variables on the system can be obtained [24]. Once each dependency is clear, device performance can be fully utilized under carefully chosen conditions.

In this study, the a-Si:H film was used as a photosensitive layer, and the electrochemical behavior of the a-Si:H photosensitive chip was studied by EIS. It was found that the resistance of the photosensitive chip decreased with increasing AC frequency. Then the volt-ampere characteristics of the chip were measured. The photoconductive threshold voltage of the a-Si:H photosensitive chip was about 0.7 V, and the DC bias increased the light-dark resistance differences in the chip. Finally, the feasibility of improving the detection performance of the system was verified by recording the electrical signals of cardiomyocytes with and without an external power supply on the photosensitive chip. Therefore, the AC or DC signals applied to the photosensitive chip improved the signal-to-noise ratio of detection and enhanced the small signal detection capability of the device. This study creates favorable working conditions for a-Si:H photosensitive chips to detect weak bioelectrical signals, enhancing their practicality in cellular electrophysiological detection and promoting research into applications of these chips as high-performance cell–material interfaces.

## 2. Materials and Methods

### 2.1. Chip Preparation

a-Si:H has been widely studied as a nanoscale thin film semiconductor, with a high light absorption coefficient, low-cost mass fabrication, and good biocompatibility [25,26]. It has been widely used in large-area electronic devices [27] such as biosensors [28] and thin film transistors [29]. In the context of nanomanufacturing, precise control over the deposition process and characterization of film properties at the nanoscale are crucial for optimizing device performance.

In this study, a 50 nm *n*-type doped a-Si:H layer and a 400 nm intrinsic a-Si:H photosensitive layer were sequentially prepared by PECVD on an ITO conductive glass. A 3 cm × 3 cm ITO conductive glass was thoroughly cleaned, dried with N_2_, and then placed in the PECVD reaction chamber. The chamber was preheated to 250 °C and purged with Ar plasma for 5 min. Subsequently, an *n*-type a-Si:H layer (50 nm) was deposited at 250 °C under 0.64 Torr with gas flows of 3 sccm SiH_4_, 60 sccm H_2_, and 11 sccm PH_3_, using an RF power of 5 W. After the *n*-layer deposition, PH_3_ was shut off, and H_2_ plasma cleaning was conducted for 5 min to eliminate residual phosphorus dopants. The intrinsic a-Si:H layer (400 nm) was then deposited by adjusting parameters to 1 Torr and 7 W with the gas flows of 20 sccm SiH_4_ and 100 sccm H_2_. Finally, the sample was annealed in situ at 200 °C for 30 min under H_2_ atmosphere to relieve interfacial stress and optimize interface bonding strength. Before depositing the photosensitive films, the edges of the ITO conductive glass were masked to reserve an exposed ITO strip (≈3 mm in width) as the measurement electrode for external circuit connection. The intrinsic layer was the main light-absorbing layer, which harvested light to create electron-hole pairs [13], thereby creating local virtual conductive pathways. The doped layer optimized the electrical contact between the intrinsic layer and ITO.

### 2.2. Electrochemical Characterization

The study of solar cells is usually conducted using EIS [30], a well-established tool for studying electrocatalytic reactions by investigating charge transport and transfer processes [31]. We applied a small amplitude AC signal (over a wide frequency range) superimposed on an appropriate DC voltage to the battery. The relative phase and amplitude of AC and voltage were measured, and the information about the sample was extracted from model analysis [32].

**Figure 1:**
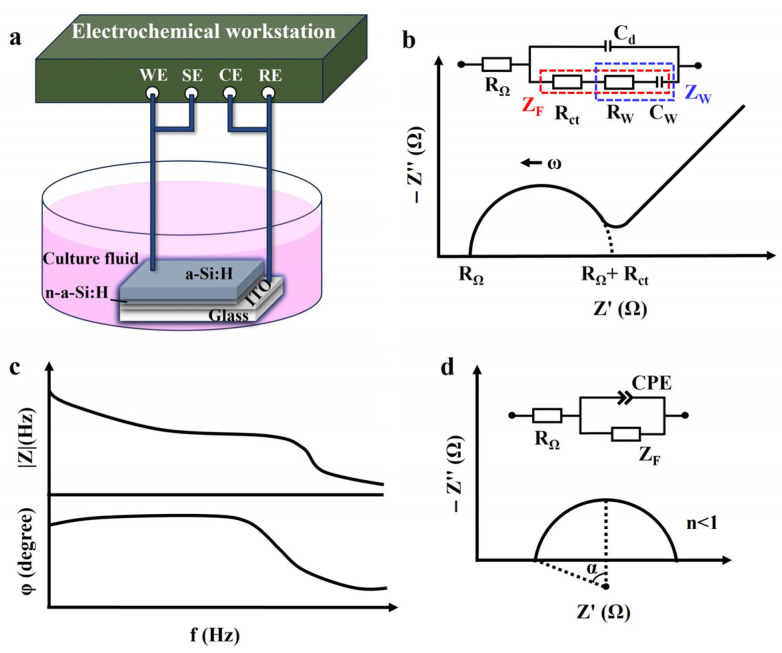
Electrochemical characterization. (**a**) Schematic of an electrochemical system with the two-electrode set-up. (**b**) Randles equivalent circuit and Nyquist plot in complex impedance plane. (**c**) Bode plot. (**d**) Non-ideal behavior of capacitance, shown as a depressed semicircle in the Nyquist plot (α represents the semicircle’s depression angle).

The EIS of the a-Si:H photosensitive chip was measured using an electrochemical workstation (PARSTAT 4000A; 0 V DC voltage, 10 mV AC voltage, 30 times per second). A two-electrode cell system was used in this study, as shown in Figure 1a. The photosensitive chip was placed in DMEM+10% FBS (Dulbecco’s modified Eagle medium with 10% fetal bovine serum) cell culture fluid. The a-Si:H photosensitive layer was used as the working electrode, and ITO was used as the reference electrode. All of the electrochemical characterization was conducted at room temperature.

To illustrate a specific phenomenon or feature, graphical methods were used to interpret and visualize impedance data. A Nyquist plot (-*Z*″ vs. *Z*’) depicts the relationship between the imaginary part of impedance *Z*″ (plotted on the *y*-axis) and the real part *Z*’ (plotted on the *x*-axis). It represents the impedance on the complex plane with frequency as the parameter and is used to describe the kinetic behavior and mass transfer [33], as shown in Figure 1b. The semicircle region corresponds to a high-frequency part, representing the charge transfer resistance and double layer capacitance at the electrode/electrolyte interface. The linear area corresponds to a low-frequency part, signifying the diffusion-limited Warburg impedance behavior [34]. A Bode plot was used to demonstrate frequency response characteristics (modulus and phase of impedance versus frequency) of the electrochemical system, as shown in Figure 1c. These are usually used for preliminary analysis of chemical systems to identify elementary processes in the mechanism [33].

### 2.3. Equivalent Circuit Model

EIS data are used to estimate the electrical components of an ECM and its combined forms to analyze the impedance behavior of a system [35]. The Warburg–Randles ECM combines the Warburg impedance and the Randles electrolytic cell model. This model is commonly used to describe complex reactions on an electrode surface, including diffusion and charge transport processes, as well as ionization and electrochemical reactions in solution. The inset of Figure 1b shows the Warburg–Randles ECM [34] of a typical Nyquist plot, including the ohmic resistance *R_Ω_* (encompassing the electrolyte resistance, electrode bulk resistance, and contact resistance), reflecting all frequency-independent purely resistive losses in the system, the Faraday impedance *Z_F_* (impedance of the electron-transfer process), and the electrical double-layer capacitance *C_d_* (impedance of the non-Faraday process). The value of *R_Ω_* can be obtained directly from the plot, which is the real part of the impedance at the starting point of the high-frequency semicircle [32]. The electrochemical impedance *Z*(*ω*) can be expressed as a combination of electrical impedance containing resistance, capacitance, or inductance and Faraday impedance [36]:

(1) $$Z\left(\omega \right)=\frac{\tilde{V}\left(\omega \right)}{\tilde{I}\left(\omega \right)}\;=\left|\frac{\tilde{V}\left(\omega \right)}{\tilde{I}\left(\omega \right)}\right|\left(\cos\phi \left(\omega \right)+j\sin\phi \left(\omega \right)\right)\;=\left|Z\left(\omega \right)\right|\left(\cos\phi \left(\omega \right)+j\sin\phi \left(\omega \right)\right)\;=Z^{\prime }+jZ\prime \prime$$

(2) $$j={\left(-1\right)}^{1/2}$$

where the variables $\tilde{V}\left(\omega \right)$ and $\tilde{I}\left(\omega \right)$ are phasors, which are used to describe the amplitude and phase of a sinusoidal function. *ω* is the angular frequency, *ϕ*(ω) is the phase shift between the voltage and the current signal, |*Z*(*ω*)| is the amplitude ratio of voltage and current, *Z*’ denotes the real impedance, *Z*″ denotes the imaginary impedance, and *j* is the imaginary unit. The electrochemical impedance represents a complex number that varies with frequency.

The Faraday impedance *Z_F_* is defined as [34]:

(3) $$Z_{F}=R_{ct}+Z_{W}$$

where *R_ct_* is the charge-transfer resistance, which refers to the resistance of the electron transfer process at the electrode/electrolyte interface and reflects the rate of the electrochemical reaction [37]. Its value corresponds to the real-axis intercept (*Z*’) at the low-frequency end of the semicircle [32]. *Z_W_* is a straight line with a 45° slope in the Nyquist plot, representing the Warburg behavior. The Warburg impedance modeling simulates a semi-infinite linear diffusion of the charged particles, which can diffuse infinitely to a large planar electrode [38].

Notably, if the high-frequency part of the Nyquist plot is a depressed semicircle, as shown in Figure 1d, it indicates a non-Debye relaxation type [39]. In this case, a constant phase element (CPE) is introduced instead of a pure capacitor to match the non-ideal behavior of capacitance [40]. Factors contributing to the non-ideal behavior of the system include inhomogeneity of the electrode material, gradient concentrations, and surface roughness [41]. The impedance of CPE is given by Equation (4) [42]:

(4) $$Z_{CPE}=\frac{1}{Q{\left(j\omega \right)}^{n}}$$

The real part of $Z_{CPE}$ exhibits resistive behavior, decreasing with increasing frequency. The imaginary part exhibits capacitive behavior, and its magnitude decreases with increasing frequency. The impedance of the semicircle is [33]:

(5) $$Z\left(\omega \right)=R_{\Omega }+\frac{Z_{F}}{1+Z_{F}{\left(j\omega \right)}^{n}Q}$$

where *Q* and *n* represent a proportional factor and an empirical exponent (0 ≤ *n* ≤ 1), respectively. *Q* is a constant and proportional to the capacitance magnitude induced by CPE. *n* is a dimensionless parameter that indicates the degree of deviation from the ideal Debye model, in which *n* = 0 means ideal resistive behavior and *n* = 1 means the ideal capacitive behavior [40].

The Warburg impedance in the low-frequency region is expressed as [43]:

(6) ZW=σω−12−jσω−12

where σ is determined by the concentrations and diffusion coefficients of oxides and reducing species.

### 2.4. Fitting the Electrochemical Impedance Spectrum

In order to obtain information related to the electrical properties of the sample, its impedance response was fitted to an ECM composed of common electrical components and other specific circuit components [44]. For a model to be meaningful, its components should accurately describe the electrochemical phenomena occurring in the system [39]. The fitting process was divided into two steps. Firstly, selecting a simple equivalent circuit that may include several resistors, capacitors, or CPEs. This was followed by computer-software-assisted fitting to match the parameters of each circuit element [44].

ZView software was used to plot and analyze impedance data and provide equivalent circuit modeling [45]. In this paper, the experimental data were fitted based on a complex nonlinear least-squares (CNLS) algorithm in the ZView software (version 3.1, Scribner Associates Inc., Southern Pines, NC, USA) [42]. The electrical parameters were extracted by effectively fitting the experimental data using an equivalent circuit [46].

### 2.5. Characterization of Volt-Ampere Characteristics

Volt-ampere characteristics were performed using a digital source meter (Keithley 2400, Keithley Instruments, Cleveland, OH, USA) and a probe station (Ecopia EPS-300, Ecopia, Anyang, South Korea). A laser with a wavelength of 671 nm was selected to irradiate the surface of the photosensitive chip. During the test, the two probes were in contact with the ITO film and the a-Si:H film, respectively. It was necessary to ensure that the test points of the probes were located in the same place before and after illumination. The scanning voltage range was set to −8 V–8 V and the scanning step size to 0.053 V.

### 2.6. Analog Experiments

The photosensitive chip was placed in the DMEM+10% FBS cell culture fluid. A signal generator (Tektronix AFG3022C, Tektronix, Beaverton, OR, USA) was used to provide AC signals or DC signals to the photosensitive chip. A sinusoidal signal with a frequency of 1 Hz–100 KHz and an amplitude of 0–1 V was selected as the AC input. The DC voltage was 0–2 V. A signal was applied to the a-Si:H layer of the photosensitive chip, and the electrical response at both ends of the chip was captured. The bright state was provided by a projection system (P150G, AMOOWA, Shenzhen, China).

### 2.7. Cellular Electrical Signal Recordings

Based on the photoelectric properties of the photosensitive chip, virtual electrodes were generated in the irradiated area as local conductive pathways for recording the electrical signals of cells. This has been reported in our previous work for recording electrical signals of cardiomyocytes [11]. The details are described in the supplementary information. To verify whether the AC excitation introduces artifacts, the system output before and after the AC excitation under cell-free conditions was set as the control group. Primary cardiomyocytes were extracted from 1- to 3-day-old newborn Sprague–Dawley rats and cultured on the photosensitive chip. The Sprague–Dawley rats were obtained from Changchun Yisi Laboratory Animal Technology Co., Ltd (Changchun, China). The photosensitive chip culture dishes were maintained in an incubator at 37 °C with 5% CO_2_. The culture fluid was replaced every two days. After 2–3 days of incubation, the morphology and contractile activity of cardiomyocytes were observed using an optical inverted microscope (Nikon Eclipse Ti-S, Tokyo, Japan). At this stage, most cardiomyocytes exhibited regular spontaneous contractions. Cardiomyocytes with a favorable physiological status were selected as target cells for subsequent experiments. The light-induced electrode with a size of 20 µm was moved to the target cell position to achieve precise alignment with the individual cell. The chip was connected to an external acquisition device (acquisition card: NI PCI-4462) for recording electrical signals of individual cells.

The AC voltage applied to the cell should be lower than the resting potential of the cell [32]. In this study, the cells tested were cardiomyocytes with a resting potential of approximately −70–90 mV [47]. In addition, since the volt-ampere characteristics of semiconductor multi-layer structures were nonlinear, the AC amplitude applied to the chip must be sufficiently small to avoid measurement-induced biasing of the junction [32].

### 2.8. Ethics Statement

Animal primary cell lines included in this study were approved as part of this study protocol. This animal study was approved by the Ethical Committee of International Research Centre for Nano Handling and Manufacturing of China (No. CNM-20240926-01). In all experiments, the relevant ethical standards, regulations, and laws in the “Regulations on the Administration of Laboratory Animals” in China were strictly observed (No.676, China, The State Council, 2017).

## 3. Results and Discussion

### 3.1. Electrical Characteristics and Fitting Results

**Figure 2:**
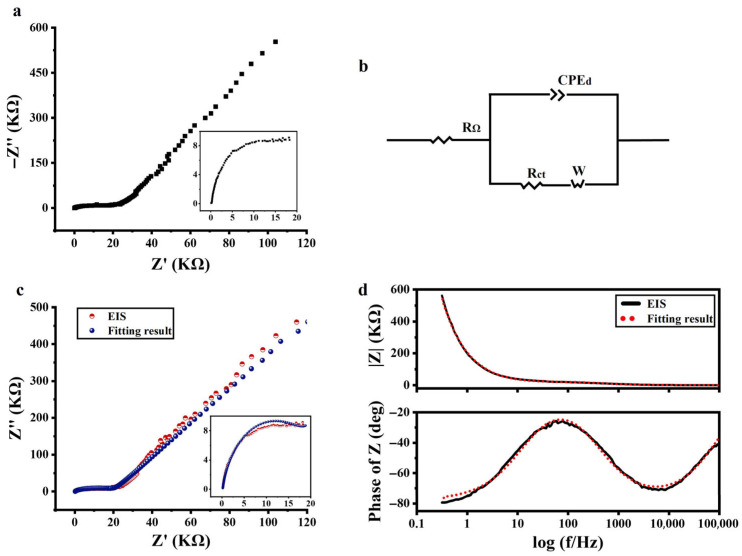
Electrical characteristics of the a-Si:H photosensitive chip. (**a**) Nyquist plot of EIS (−*Z*″ vs. *Z*’). (**b**) Equivalent circuit used to fit the EIS. (**c**) Nyquist plots of EIS measurement (red dots) and fitting (blue dots). (**d**) Bode plot of EIS (black solid line) and respective fitting data (red dots) (variation in impedance modulus (|*Z*|) and phase vs. frequency).

Figure 2a shows the Nyquist plot of the a-Si:H photosensitive chip, with the high-frequency portion of the spectrum magnified on the right. The curve displays a depressed arc (not centered on the real axis), indicating that the system under test is of a non-Debye relaxation type. Considering the EIS measurements results and the previous introduction (basic characteristics of EIS and ECMs), it was determined that the Warburg–Randles combined ECM was used to describe the a-Si:H photosensitive chip. CPE was used to replace a pure capacitor. The equivalent circuit of the a-Si:H photosensitive chip used to fit the measured impedance data is shown in Figure 2b.

The experimental data were fitted using the ZView software to assign the appropriate physical or structural elements to each component of the equivalent circuit. The Nyquist plot and Bode plot of the experimental data, as well as their fitting results, are shown in Figure 2c,d. It can be seen that the resulting fitted curve has good consistency with the experimental data. It demonstrated that the equivalent circuit in Figure 2b was available for electrical modeling of a-Si:H photosensitive chips and could be explained by existing theories. The best-fitting values for equivalent circuit elements are detailed in the supplementary information (Reference [46] are cited in the supplementary information).

In the Bode plot, the impedance mode (|*Z*|) was higher at lower frequencies, then decreased with the increasing in frequency, and varied less at higher frequencies. This was a result of the semiconductor behavior of the photosensitive chip, namely that the conductivity increased with frequency. The same phenomenon was observed in the Nyquist plot.

### 3.2. Equivalent Circuit of the System and Simulation

**Figure 3:**
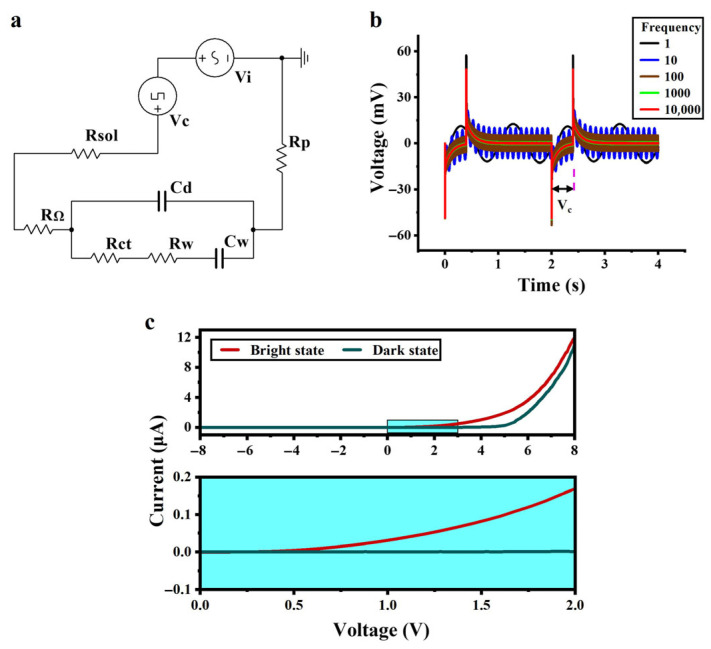
Circuit simulation and volt-ampere characteristics. (**a**) Equivalent circuit of the system containing an analog signal source for a cell and an applied AC signal. (**b**) Circuit simulation results. (**c**) Volt-ampere characteristics of the a-Si:H photosensitive chip in the dark and bright states.

The equivalent circuit of the system was drawn on the basis of the equivalent circuit of the a-Si:H photosensitive chip and the fitted parameters, as shown in Figure 3a. V_c_ simulates an input of a cellular electrical signal (modulating signal) with a voltage of 50 mV and a duty cycle of 20%. V_i_ is an applied AC signal (carrier signal) with a voltage of 10 mV. R_sol_ is the solution resistance, and R_p_ is the external protection resistance. With various frequency inputs of the carrier signal V_i_, the simulation results are shown in Figure 3b. It was observed that as the frequency of the applied AC signal increased, the background noise of the whole system decreased. The signal-to-noise ratio was improved so that small signals could be extracted more efficiently.

### 3.3. Volt-Ampere Characteristics

Figure 3c shows the volt-ampere curve of the a-Si:H photosensitive chip. The current of the a-Si:H photosensitive chip is significantly higher in the bright state than in the dark state, indicating an increase in carrier concentration and improved charge transport ability, which is consistent with the photosensitivity of the material. When the applied voltage exceeds 0.7 V, the bright state current exhibits a remarkable jump, thus determining its photoconductivity threshold voltage to be 0.7 V. After exceeding this threshold, the current difference between the bright and dark states begins to increase. This is because the electric field promotes the transport of photogenerated carriers while suppressing the random diffusion of dark state leakage current, shifting the chip from a “low-sensitivity response region” to a “high-sensitivity response region”.

In terms of carrier transport mechanisms, the electric field reduces the energy required for electrons to escape from traps. This phenomenon originates from the defect-mediated Poole–Frenkel (PF) effect: under strong electric fields, the trap barrier is lowered through field-assisted thermal excitation, enabling trapped electrons to escape into the conduction band via thermionic emission, promoting carrier transport [48].

### 3.4. Effect of AC Signals on the a-Si:H Photosensitive Chip

**Figure 4:**
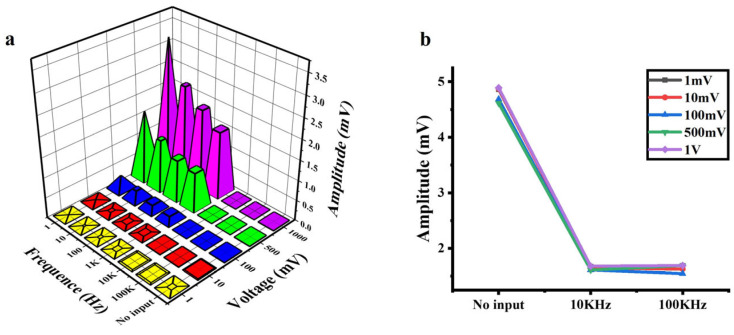
Output signals of the a-Si:H photosensitive chip applied with AC signals. (**a**) Output signals vs. the frequency and amplitude of AC input signals. (**b**) Background noise at no input and 10 KHz/100 KHz AC input (at various amplitudes).

Applying an AC signal at different frequencies and amplitudes to the photosensitive chip, the output voltage at both ends of the chip is shown in Figure 4a. With the constant amplitude of the input signal, the amplitude of the output signal decreased as the frequency of the input signal increased (1 Hz–100 KHz). This frequency-dependent attenuation is consistent across all input amplitudes. Notably, the amplitude of output signals decreased significantly at frequencies up to 10 KHz. The amplification results with no input (no electrical signals applied) and the AC input with the frequencies of 10 KHz and 100 KHz (at various amplitudes) are shown in Figure 4b. Comparing the outputs at 10 KHz and 100 KHz, the effects of the amplitude and frequency of the AC input can be ignored. Importantly, the amplitude of the output signal at 10 KHz is lower than that without input, demonstrating effective background noise suppression via high-frequency excitation.

When the signal is weak, the noise of the cell–material interface may affect the accuracy of detection. The background noises of a-Si:H photosensitive films mainly include flicker noise (1/f noise), thermal noise, and shot noise [49,50,51]. Flicker noise originates from the random trapping and release of charge carriers by defect states, which mainly appears in the low-frequency region below about 1 KHz, and the power spectral density decays with increasing frequency [52]. Thermal noise is caused by the thermal motion of electrons in resistive materials, exhibiting a frequency-independent power spectral density (white noise) [53]. Shot noise results from the discrete nature of carrier crossing the PN junction barrier. Its frequency spectrum is analogous to thermal noise, remaining constant over a wide frequency range and thus is also classified as white noise [53]. The core mechanism of high-frequency noise reduction is to suppress the noise components related to frequency. High-frequency AC modulation (>10 kHz) drives defect states into dynamic equilibrium, attenuating 1/f noise while spectrally flat thermal/shot noise remains unaltered.

### 3.5. Effect of DC Bias on the a-Si:H Photosensitive Chip

**Figure 5:**
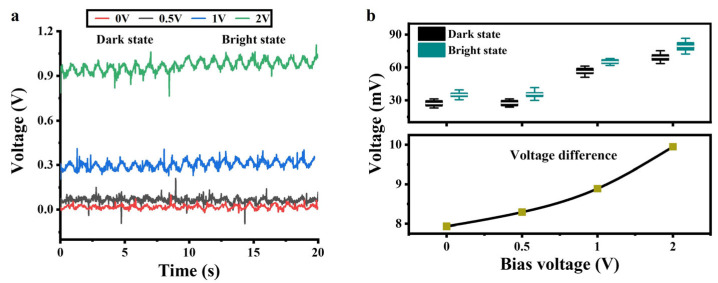
Output signals of the a-Si:H photosensitive chip applied with a DC bias. (**a**) Output signals vs. the DC bias in the dark and bright states. (**b**) Voltage statistics in the light and dark states of (**a**).

Based on the volt-ampere characteristics of the a-Si:H photosensitive chip, we provided a DC bias to the chip to improve the detection performance. A sinusoidal signal with a frequency of 1 Hz and an amplitude of ±25 mV was applied to the photosensitive chip to simulate electrical signals in cardiomyocytes. Four sets of DC signals were provided to the chip, respectively. Figure 5a shows the output signals of the photosensitive chip in the bright and dark states. The statistics represent the amplitudes of the output signals at each DC bias in the light and dark states, as shown at the top of Figure 5b. The amplitude differences between the output signals in the dark and light states are shown at the bottom of Figure 5b. As the DC bias increased, the voltage differences became larger. The volt-ampere characteristics of the a-Si:H photosensitive chip in Figure 3c show that the bright state conductivity increases nearly linearly with the bias voltage (0.7–2 V), while the dark state conductivity remains almost unchanged (depending on thermally excited carriers). This results in an increase in the resistance difference between bright and dark states.

### 3.6. Electrical Recordings of Cardiomyocytes

Based on the above results and analysis, we proposed to apply an AC signal or a DC bias signal to the a-Si:H photosensitive chip to improve the performance in detecting weak bioelectric signals. The premise is that the applied signals have no effect on the cells. The DC voltage may generate electrical stimulation on the cell membrane, affecting cell viability and even causing damage. In contrast, AC signals, particularly at high frequencies, may mitigate this risk, but it is necessary to determine whether the frequency range interferes with the activity of cells. During light irradiation, when a 2 V DC voltage was applied to the chip, bubbles were generated, and no breakdown of the chip was observed, indicating the occurrence of water electrolysis. To avoid affecting the normal physiological activities of cells, the DC voltage was selected to be 0–1 V. The pulsation state of the cardiomyocytes was observed from a macroscopic viewpoint to verify whether the cells were stimulated. Detailed results are provided in the supplementary information. Compared with no input, there was no significant difference in the number of cardiomyocyte pulsations during the applied signal. Therefore, AC signals with an amplitude of 10 mV at the frequencies of 1 Hz–100 KHz or DC biases in the range of 0–1 V applied to the chip did not stimulate cardiomyocytes.

The selection of AC frequency should not overlap with the biological signal frequency band (approximately 0.1–5 Hz for cardiomyocyte action potentials) to avoid interference. On the basis of the laws presented in the aforementioned theories, circuit simulation of the system and analog experiments, it was determined to apply a 10 mV AC signal at 10 KHz or a 1 V DC bias to the a-Si:H photosensitive chip on which cardiomyocytes were cultured.

**Figure 6:**
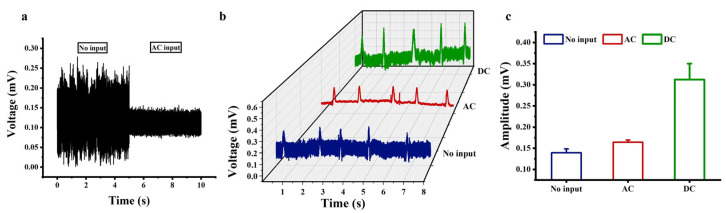
Electrical recordings of cardiomyocytes based on the a-Si:H photosensitive chip. (**a**) System output before and after the AC excitation under cell-free conditions. (**b**) Electrical signals recorded by the photosensitive chip at no input, a 10 mV AC signal at 10 KHz, or a 1 V DC bias. (**c**) Amplitudes of electrical signals (mean ± SD).

Under cell-free conditions, the system output before and after the AC excitation application is shown in Figure 6a. Without the AC excitation, the baseline noise was approximately 170 μV. When applying 10 mV AC excitation at 10 kHz, the baseline noise significantly decreased to about 30 μV, with no periodic peaks or spectral anomalies observed in the output signal. This demonstrates that the AC excitation effectively suppresses system noise without introducing artifacts. Figure 6b shows the electrical recordings from the cardiomyocytes cultured on the a-Si:H photosensitive chip. There were three states, namely no input, 10 mV AC input at 10 kHz to the photosensitive chip, or 1 V DC input to the photosensitive chip. Comparing the electrical signals without input and AC input in Figure 6b shows that applying an AC signal reduced the background noise of the photosensitive chip electrical recording system by approximately 85.1%. Therefore, applying a 10 mV AC signal at 10 KHz to the photosensitive chip significantly improved the signal-to-noise ratio of the detection signal, making the cellular electrical signal more prominent. Comparing the electrical signals without input and DC input in Figure 6b shows that applying a 1 V DC bias to the photosensitive chip increased the amplitude of the cellular electrical signals by approximately 142.7%. The amplitudes of the cellular electrical signals were extracted, as shown in Figure 6c. Therefore, the electrical signals recorded by the photosensitive chip with AC or DC signals applied were significantly improved over those recorded with no input. The noise reduction under the high-frequency AC excitation is related to the suppression of 1/f noise. After applying a 1 V DC bias, the chip is “pre-activated” to the high-sensitivity working regime, resulting in a more prominent modulation effect of the light-induced electrode on the bioelectric signal. Due to the enhanced motion of carriers, the current difference between the bright and dark states becomes more significant, making it easier for the chip to capture current changes caused by weak bioelectric signals. These results validate the feasibility of improving the detection performance of the a-Si:H photosensitive chip by providing external excitation at both ends of the photosensitive chip.

## 4. Conclusions

This study establishes an optimized operational condition for a-Si:H photosensitive chips to significantly enhance the detection of weak bioelectrical signals. Through systematic analysis of EIS and volt-ampere characteristics, we revealed two critical operational mechanisms that maximize the chip’s performance: the high-frequency AC excitation suppresses the background noise by utilizing frequency-dependent impedance reduction; the DC bias amplifies the signal contrast by increasing the difference in light and dark resistance. This work overcomes the inherent limitations in the a-Si:H photosensitive biological interface through the AC/DC input strategy, and significantly improves the signal-to-noise ratio of recorded cellular electrical signals under the optimized AC (10 mV, 10 kHz) or DC (1 V) excitation compared to direct detection. By exploiting inherent optoelectronic properties for the on-chip noise suppression and signal amplification, we provide favorable working conditions for a-Si:H photosensitive chips, maximizing the advantages of the chip. We show that the improved detection performance of the bioelectric recording interface based on light-induced electrodes provides a new perspective for studying cellular electrical signals and expands the application of photosensitive materials in cell–material interfaces.

## Data Availability

The original contributions presented in this study are included in the article/supplementary material. Further inquiries can be directed to the corresponding author(s).

## Supplementary Materials

The following supporting information can be downloaded at: https://www.mdpi.com/article/10.3390/s25175255/s1, Figure S1: Schematic structure of the a-Si:H photosensitive chip with cells cultured on it; Table S1: The R, CPE, and W extract parameters for a-Si:H film sample; Table S2: Number of cardiomyocyte pulsation in 10 s at the different frequencies of 10 mV AC signals; Table S3: Number of cardiomyocyte pulsation in 10 s at the DC biases in the range of 0–1 V.

## Abbreviations

The following abbreviations are used in this manuscript:

a-Si:H	hydrogenated amorphous silicon
EIS	electrochemical impedance spectroscopy
ECM	equivalent circuit model
AC	alternating current
DC	direct current
MEA	microelectrode array
PECVD	plasma-enhanced chemical vapor deposition
ITO	indium tin oxide
DMEM+10% FBS	Dulbecco’s modified Eagle medium with 10% fetal bovine serum
CPE	constant phase element
CNLS	complex nonlinear least-squares
PF	Poole–Frenkel
1/f noise	flicker noise
