# Supplementary material for: Cellular Signal Detection by Hydrogenated Amorphous Silicon Photosensitive Chip with Electroexcitation
Source: Sensors (Basel). 2025 Aug 23;25(17):5255. doi: 10.3390/s25175255 (PMC12431506; doi:10.3390/s25175255)
Supplement: Supplementary file 1 [file sensors-25-05255-s001.zip › sensors-3766156-supplementary.pdf]

Cellular Signal Detection by Hydrogenated Amorphous Silicon Photosensitive Chip with Electroexcitation

Design and preparation of light-induced electrode

Hydrogenated amorphous silicon (a-Si:H) is extremely sensitive to light. Light-induced electrodes were prepared based on the photoconductive properties of the a-Si:H photosensitive chip with current conduction in the presence of light and current cut-off in the absence of light. For a-Si:H, under the standard AM1.5 illumination (100 W/cm^2 at 25°C), the photoconductivity is >10^{-5} (Ω·cm)$^{-1}$, and the photosensitivity is in the order of 10^5 -10^6 [13]. Therefore, a-Si:H was chosen as the photosensitive material.

The corresponding light-induced electrodes were formed using the light pattern projected onto the photosensitive chip. When the light pattern was projected on the surface of a-Si:H, a large number of photogenerated carriers were generated in the illuminated area due to the photoconductive characteristics of a-Si:H material [14]. In this case, the resistance of the illuminated area rapidly decreased and the conductivity increased. Therefore, electrodes defined by light patterns, namely light-induced electrodes, were generated on the chip.

The computer was used to design different light patterns, and the digital projector (P150G, AMOOWA, China) projected the designed light patterns onto the a-Si:H photosensitive chip through two lenses and an objective lens. The functions of the lenses and the objective lens were to converge the light intensities and adjust the light patterns to the suitable size for measuring the electrical signals of individual cells.

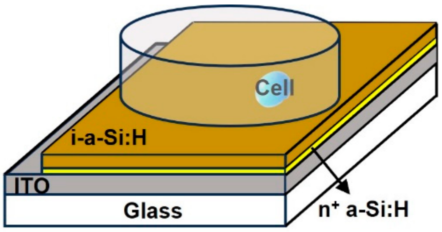

Figure S1. Schematic structure of the a-Si:H photosensitive chip with cells cultured on it

Figure S1 shows the structure of the a-Si:H photosensitive chip with cells cultured on it. The size of the chip was 3 cm × 3 cm.

Cellular electrical signals detection

Traditional MEA was recognized as a standard tool for measuring extracellular potentials. Virtual light-induced electrodes were used instead of the physical electrodes of traditional MEA for the detection of cellular electrical signals. The designed light pattern was emitted by a projection device and projected onto the photosensitive chip after passing through a series of optical devices (30-μm light line). The light pattern and cells were observed by a computer connected to an optical upright microscope and a CCD, and then the light pattern was moved to the target cells. The virtual electrodes were used as the working electrode and the electrical signals were derived from ITO. The conductivity probe placed in culture solution served as the reference electrode.

Best-fitting values for equivalent circuit elements

The best-fitting values for equivalent circuit elements of the a-Si:H photosensitive chip in Fig. 2b were summarized in table S1.

Table S1. The R, CPE, and W extract parameters for a-Si:H film sample

Element	Value	Error %
R_Ω	178.2	1.8749
CPEd-T	$5.3559e^{-8}$	2.8587
CPEd-P	0.8749	0.31573
Rct	22019	0.88451
W-R	$6.5438e^{6}$	6.774
W-T	8.428	7.7106
W-P	0.86457	0.3506
Chi-Squared	0.0029528	

Where, the parameter CPE-T is the grain boundary capacitance (constant phase elements for grain boundary), and CPE-P represents a dimensionless identity denoting the degree of deviation from the ideal behavior [46]. Parameters W-R, W-T and W-P represent the resistive part, the time part and the exponential factor of Warburg impedance, respectively.

Cardiomyocytes pulsation videos

10 mV AC signals were applied to the photosensitive chip (divided into no input, 1 Hz, 10 Hz, 100 Hz, 1 KHz, 10 KHz, 100 KHz, and no input). Each frequency input lasted for 30 seconds. The number of cellular pulsations for 10 s in the middle of each frequency was recorded separately, and the results are shown in Table S2. Similarly, DC signals were applied to the photosensitive chip (divided into no input, 10 mV, 50 mV, 100 mV, 500 mV, 1 V, and no input). Each amplitude input lasted for 30 seconds. The number of cellular pulsations for 10 s in the middle of each amplitude was recorded separately, and the results are shown in Table S3.

Table S2. Number of cardiomyocyte pulsation in 10 s at the different frequencies of 10 mV AC signals

No input	1 Hz	10 Hz	100 Hz	1 KHz	10 KHz	100 KHz	No input
8	8	8	9	9	8	8	8

Table S3. Number of cardiomyocyte pulsation in 10 s at the DC biases in the range of 0-1 V

No input	10 mV	50 mV	100 mV	500 mV	1 V	No input
11	12	11	11	12	10	10
